# Antioxidant, Anti-Inflammatory, and Inhibition of Acetylcholinesterase Potentials of *Cassia timoriensis* DC. Flowers

**DOI:** 10.3390/molecules26092594

**Published:** 2021-04-29

**Authors:** Maram B. Alhawarri, Roza Dianita, Khairul Niza Abd Razak, Suriani Mohamad, Toshihiko Nogawa, Habibah A. Wahab

**Affiliations:** 1 School of Pharmaceutical Sciences, Universiti Sains Malaysia, Minden 11800, Penang, Malaysia; maram.alhawarri@gmail.com (M.B.A.); niza@usm.my (K.N.A.R.); suriani@usm.my (S.M.); 2 USM-RIKEN Centre for Aging Science (URICAS), Universiti Sains Malaysia, Minden 11800, Penang, Malaysia; nogawat@riken.jp; 3 Chemical Biology Research Group, RIKEN Centre for Sustainable Resource Science, 2-1 Hirosawa, Wako, Saitama 351-0198, Japan

**Keywords:** *Cassia timoriensis*, antioxidant, anti-inflammatory, acetylcholinesterase, Alzheimer

## Abstract

Despite being widely used traditionally as a general tonic, especially in South East Asia, scientific research on *Cassia timoriensis*, remains scarce. In this study, the aim was to evaluate the in vitro activities for acetylcholinesterase (AChE) inhibitory potential, radical scavenging ability, and the anti-inflammatory properties of different extracts of *C. timoriensis* flowers using Ellman’s assay, a DPPH assay, and an albumin denaturation assay, respectively. With the exception of the acetylcholinesterase activity, to the best of our knowledge, these activities were reported for the first time for *C. timoriensis* flowers. The phytochemical analysis confirmed the existence of tannins, flavonoids, saponins, terpenoids, and steroids in the *C. timoriensis* flower extracts. The ethyl acetate extract possessed the highest phenolic and flavonoid contents (527.43 ± 5.83 mg GAE/g DW and 851.83 ± 10.08 mg QE/g DW, respectively) as compared to the other extracts. In addition, the ethyl acetate and methanol extracts exhibited the highest antioxidant (IC_50_ 20.12 ± 0.12 and 34.48 ± 0.07 µg/mL, respectively), anti-inflammatory (92.50 ± 1.38 and 92.22 ± 1.09, respectively), and anti-AChE (IC_50_ 6.91 ± 0.38 and 6.40 ± 0.27 µg/mL, respectively) activities. These results suggest that ethyl acetate and methanol extracts may contain bioactive compounds that can control neurodegenerative disorders, including Alzheimer’s disease, through high antioxidant, anti-inflammatory, and anti-AChE activities.

## 1. Introduction

Plants provide a significant source of bioactive compounds, such as phenolics, terpenoids, essential oils, sterols, alkaloids, polysaccharides, tannins, and anthocyanins [1]. Investigation of the biological activities of medicinal plants, particularly antioxidants, has attracted considerable interest. The antioxidant property of medicinal plant products has been shown to be primarily attributable to the phytochemical groups mentioned above [2]. These natural antioxidants prevent the destructive effects induced by oxidative damage of the free reactive oxygen species (ROS) and reactive nitrogen species (RNS) implicated in neurodegenerative diseases, such as AD [3]. 

**Figure 1:**
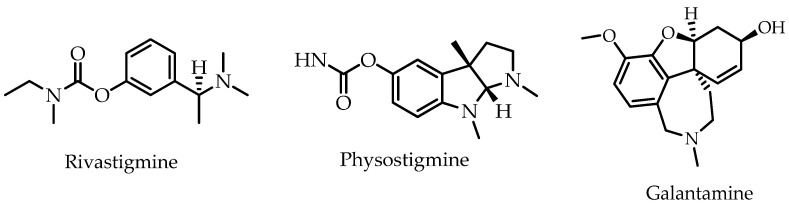
The chemical structures of rivastigmine, physostigmine, and galantamine.

Between 1981 and 2019, approximately 50% of all drugs approved worldwide were produced using or inspired by natural products [4]. The known cholinesterase inhibitor rivastigmine, used for Alzheimer’s disease (AD) treatment, is an example of a semi-synthetic drug developed based on the naturally occurring cholinesterase inhibitor physostigmine scaffold [5]. Physostigmine, an alkaloid isolated from *Physostigma venenosum*, is administered for glaucoma and myasthenia gravies treatment, but its use for AD treatment is restricted in certain countries due to the serious hepatic and cardiac side effects [6,7]. Nonetheless, galantamine (Figure 1), a pure natural product isolated from the bulbs and flowers of *Galanthus caucasicus* and *Galanthus woronowii*, is currently available on the market for the treatment of cognitive decline in mild to moderate AD [8,9].

*Cassia* is a huge genus of around 600 species of flowering trees and shrubs [10] belonging to the Leguminosae family, which comprise more than 600 genera and 18,000 species [11]. This plant family is predominantly distributed across tropical to subtropical Asian areas [12]. *Cassia timoriensis* DC. is a perennial tree or shrub, usually about 2–6 m tall. The plant is widely spread in tropical areas, particularly in South East Asian countries such as India, Sri Lanka, Thailand, Malaysia, and Indonesia [13]. A flowering plant with yellow blooms and shiny brown seedpods, *C. timoriensis* is also sometimes valued as an ornamental plant [14]. Traditionally, this *Cassia* species is used for treating toxins, scabies, itching, and skin diseases and as an anthelmintic medicine [13,15]. It is also used as a general tonic, antitumor, and for blood disorders, particularly its heartwood component, which is commonly used for menstrual blood disorder [13,14,15]. Despite its wide range of traditional uses, *C. timoriensis* has hardly been studied for its phytochemical constituents and biological activities. The first compound identified from this plant was barakol, discovered by a Thai group in 1984 [16]. Two decades later, in a screening of 20 Thai medicinal plants, an aqueous extract of *C. timoriensis* demonstrated powerful antioxidant activity through the inhibition of Heinz bodies induction [15]. Recently, after two decades of antioxidant activity study, our research group reported that *C. timoriensis* demonstrated the highest (94–97%) inhibition towards acetylcholinesterase (AChE) in a screening study for anti-cholinesterase activity of 17 methanol extracts from different parts of five *Cassia* species. Of the six isolated compounds, 3-methoxyquercetine from *C. timoriensis* leaves extract showed moderate inhibition towards AChE (IC_50_: 83.71 μM) [17]. Therefore, as a continuation of our research on biological and chemical evaluations of *C. timoriensis,* qualitative and quantitative phytochemical analyses were carried out together with in vitro studies for the acetylcholinesterase inhibitory potential, radical scavenging ability, and anti-inflammatory activity of different extracts of *C. timoriensis* flowers.

## 2. Results and Discussion

### 2.1. Phytochemical Screening of Cassia timoriensis Flowers

The phytochemical content of different extracts of *C. timoriensis* flowers was screened using standard, established protocols [18,19,20]. The screening included various secondary metabolite classes such as alkaloids, phenolics (flavonoids, coumarins, and quinones), tannins, saponins, glycosides (cardiac and anthraquinones glycosides), steroids, and terpenoids, as well as two primary metabolites (proteins and carbohydrates). The results revealed the presence of flavonoids, tannins, coumarins, steroids, and terpenoids in all extracts except for the aqueous extract. All extracts gave a negative indication for the presence of alkaloids, while anthraquinone glycosides were only detected in the ethyl acetate extract. This result provided an early indication for the probable presence of various interesting secondary metabolites in the ethyl acetate extract (Table 1). 

### 2.2. Antioxidant Capacity of Cassia timoriensis Flower Extracts

Plants are known as a natural source for antioxidants. Phenolic compounds such as phenolic acids, flavonoids, coumarins, and tannins are said to be the main compounds responsible for such activity [21]. In this study, the antioxidant capacity of different extracts of *C. timoriensis* was evaluated based on three parameters: total phenolic content (TPC), total flavonoid content (TFC), and radical scavenging activity using a 2,2-diphenyl-1-picrylhydrazyl (DPPH) assay. Total phenolic content was determined based on the Folin–Ciocalteu method and is expressed as mg gallic acid equivalent per gram of dry weight extract (mg GAE/g DW) [22]. The Folin–Ciocalteu assay is convenient, simple, precise, and reproducible and is based on the oxidation–reduction reaction involving single electron transfer (SET) from phenolic compounds to molybdenum reagent in an alkaline medium. It turns the solution from yellow to a blue complex of reduced molybdenum that can be detected spectrophotometrically at 750–765 nm. On the other hand, total flavonoid content (TFC), expressed as mg quercetin equivalent per gram of dry weight extract (mg QE/g DW), was assessed using an aluminum chloride-based colorimetric assay [23], and the scavenging ability of the extracts towards radical DPPH was also evaluated to establish their in vitro antioxidant activity [24]. Decolorization of the purple solution of DPPH to yellow indicates the reduction of DPPH following the interaction of radical DPPH with antioxidant compounds in the extract.

In general, all extracts of the *C. timoriensis* flower showed relatively high phenolic and flavonoid contents. The highest TPC and TFC values were observed in the ethyl acetate extract, followed by the methanol and hexane extracts (Table 2). The lowest TFC and TPC values were found in the aqueous extract of *C. timoriensis* flowers. This finding is in line with the phytochemical screening result, where most flavonoid and phenolic metabolites were distributed in the ethyl acetate, methanol, and hexane extracts. Similar results were reported by Kolar et al., (2018), where the flower extracts of *Cassia auriculata, Cassia italica, Cassia siamea*, and *Cassia uniflora* showed high phenolic and flavonoid contents as compared to the other parts, such as pod, stem, and leaf [25]. Although phytochemical investigations of *C. timoriensis* are still scarce, the phytochemicals of other *Cassia* species are well studied and documented [26]. Juan-Badaturuge et al. identified kaempferol-3-O-rutinoside, kaempferol, quercetin, and luteolin in *C. auriculate* as potent antioxidants through activity-guided fractionation and isolation [27]. Kaempferol and luteolin have also been isolated from *C. alata* and *C. fistula* and displayed a strong DPPH scavenging activity [28,29,30]. On the other hand, anthraquinones were found as the major phenolic compounds present in *Cassia* species [31], while emodin, aloe-emodin, rhein, and chrysophanol are widely distributed in *Cassia* species such as *C. tora* [32], *C. roxburghii* [33], *C. alata* [34,35], *C. obtuse* [36], *C. siamea* [37], and *C. angustifolia* [38]. All of these previous findings are in good agreement with our present study that demonstrates *C. timoriensis* extract to possess high contents of flavonoids and phenolic compounds. 

**Figure 2:**
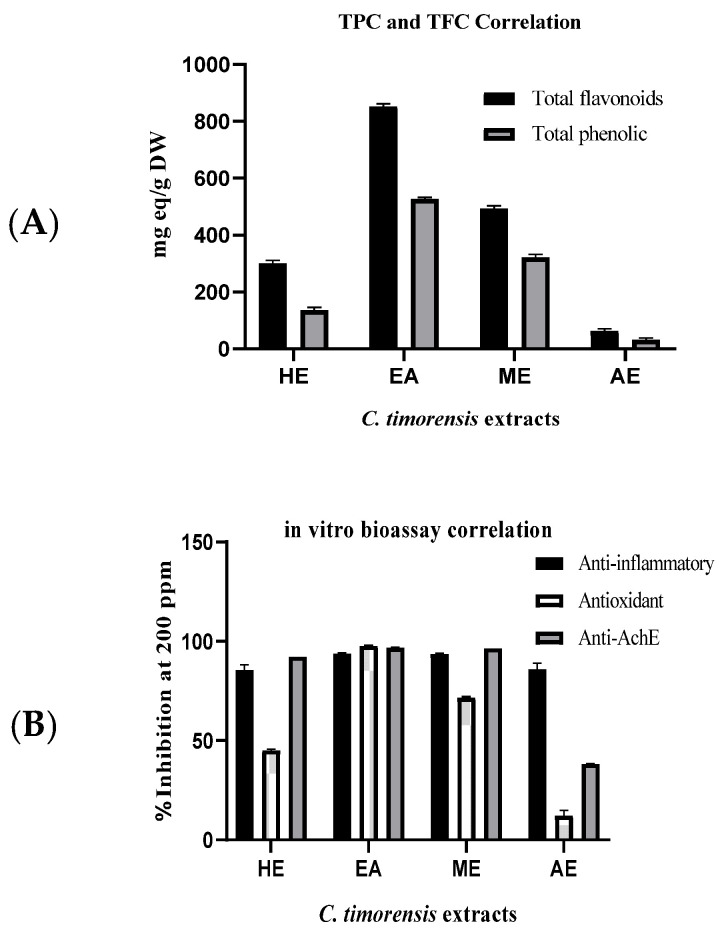
(**A**) Relationship between total phenolic content and total flavonoid content of *Cassia timoriensis* extracts. (**B**) Relationship between total phenolic content and flavonoid content with in vitro bioassays for *Cassia timoriensis* flower extracts.

The extracts of *C. timoriensis* flowers were then evaluated for their antioxidant activity. Among all of the extracts, the ethyl acetate extract possessed the highest antioxidant activity with IC_50_ 20.12 ± 0.12 µg/mL, followed by methanol, hexane, and aqueous extracts. However, the activity of the aqueous extract at 50 µg/mL was very low (<25% inhibition). Thus, the IC_50_ was not determined. The ability of the ethyl acetate extract to inhibit DPPH oxidation is comparable to the positive control, ascorbic acid, with similar percentages of inhibition at 50 µg/mL and IC_50_ values. It is postulated that the diverse phenolic content of the ethyl acetate extract may provide a wide range of proton-donating compounds that act as potent antioxidant agents via free radical inhibition or scavenger mechanisms. The results also showed a positive correlation between the TPC/TFC of the extracts and their radical DPPH scavenging activity (*R*^2^ values are 0.986 and 0.934, respectively) (Figure 2). This implies that the higher the phenolic and flavonoid compound contents are in the extract, the stronger the antioxidant activity displayed by the extract is. In addition, many studies have postulated the antioxidant mechanisms of flavonoid- or phenolic-rich plants [39,40,41]. The antioxidant behavior of phenolic compounds might be due to the activity of hydrogen or electron-donating agents in stabilizing and delocalizing the unpaired electron and their transition metal-chelating potential, especially with iron and copper [21]. The oxidation–reduction potential of these compound classes depends on the number and arrangement of the hydroxyl groups in the structure and also the replacement of the hydroxyl-contributing groups with other groups such as glycosides [21]. The findings from the DPPH scavenger assay supported the importance of the -OH group of phenolic compounds in the electron transfer reaction that is responsible for the antioxidant activity. 

Many studies have shown the potentially beneficial effect of some *Cassia* species against various chronic diseases such as cardiovascular disease [42,43,44], brain diseases [45,46], and cancers [47,48,49]. For most *Cassia* species, these natural protective effects are primarily due to the presence of pro-anthocyanidins and phenolic and flavonoid compounds [50,51]. The radical scavenging ability of the phenolic compounds is postulated to play important role in reducing oxidative stress in the body due to the presence of high levels of radical oxygen species (ROS) and radical nitrogen species (RNS). The presence of a high level of ROS and RNS is linked to many chronic immunoinflammatory and degenerative diseases [40,52,53,54]. Thus, the strong antioxidant potential of *C. timoriensis* correlates well with its traditional use as a general tonic as well as with alleviating body toxins [13]. *C. timoriensis* may aid in maintaining human well-being and preventing cell damage due to oxidative stress.

### 2.3. Anti-Inflammatory Activity of Cassia timoriensis Flower Extracts 

Inflammation is one of the body’s defense mechanisms. One of the first indicators of the inflammatory process is the denaturation of cellular proteins following tissue or cell injury, in which a series of pro-inflammatory mediators (TNF-α, interleukins, NF-κB, nitric oxide, and prostaglandins) and radical species (ROS or RNS) are released [55]. Chronic inflammation or overproduction of pro-inflammatory mediators and radical species might lead to certain chronic diseases, such as rheumatoid arthritis, diabetes, atherosclerosis, and neurodegenerative diseases [56]. 

Albumin is the most abundant protein in the blood plasma and is able to bind and transport various compounds, such as fatty acids, bilirubin, tryptophan, hormones, and a large variety of medications [57]. The chemical structure of albumin can be altered by pro-inflammatory mediators, leading to rapid clearance. Reductions in plasma albumin levels during inflammation are primarily mediated by IL-6 and TNF-α [58,59]. Non-steroidal anti-inflammatory drugs (NSAIDs), such as ibuprofen and indomethacin, have been reported to exert their anti-inflammatory function by multiple mechanisms, including stabilizing the albumin structure [60,61]. Hence, the ability to inhibit protein denaturation signifies the apparent potential for anti-inflammatory activity. In this study, the inhibition of protein denaturation by the extracts of *C. timoriensis* flower was assessed at two different concentrations. At 200 µg/mL, all *C. timoriensis* flower extracts showed high inhibitory activity against protein denaturation (>85% inhibition), which was comparable to the positive control, indomethacin (91% inhibition) (Table 3). The inhibition activities of the ethyl acetate and methanol extracts were still high at 100 µg/mL, which is a similar pattern to the positive control, indomethacin. However, the inhibition activities of both the hexane and aqueous extracts were reduced significantly at 100 µg/mL (less than 50% inhibition). The high phenolic and flavonoid contents of the ethyl acetate and methanol extracts of *C. timoriensis* flowers might account for their high anti-inflammatory activity (Figure 1). The results are in agreement with previous reports where phenolic compounds, including flavonoids, showed anti-inflammatory activity through various mechanisms [62,63]. 

Plant sterols and flavonoids have been reported as promising anti-inflammatory agents that modulate immune-inflammatory markers such as Th1/Th2 and the cytokines TNF-α, IL-1, IL-6, and IL-8 [64]. Evaluation of the anti-inflammatory activity of various species from the *Cassia* genus [46,65,66,67,68,69] has led to the identification of several potential bioactive compounds, such as cassiaindoline and rhein [70,71,72]. Specifically, stigmasterol and β-sitosterol were reported to reduce TNF-α in a cutaneous allergic response [73] and to block mast cell-derived caspace-1 and NF-κB signal pathways in atopic dermatitis-like skin lesions [73,74]. Thus, the positive indication of steroids and triterpenoids in *C. timoriensis* flower extracts in our present study as well as the fact that β-sitosterol and stigmasterol were isolated from *C. timoriensis* in our previous study [17] suggest the potential anti-inflammatory activity of *C. timoriensis*, which directly supports its traditional use for skin disorders, itching, and scabies. Further study, however, is warranted to establish the anti-inflammatory role of *C. timoriensis*.

Moreover, a number of non-antimicrobial therapeutic agents, including ibuprofen, have been found to play a role in multidrug-resistant infections such as methicillin-resistant *Staphylococcus aureus* (MRSA). Furthermore, there is an increasing interest in the efficacy of herbal products and essential oils as a health remedy for the control of drug resistance issues, which may be due to the synergistic influence of bioactive compounds [75,76]. For example, GeloMyrtol (G. PohlBoskamp, Hohenlockstedt, Germany) is a notable herbal medicine used to treat asthma and sinusitis. GeloMyrtol is extracted from a variety of essential oils provided by *Citrus limon*, *Camellia sinensis*, *Eucalyptus globulus*, and *Myrtus communis* [76]. Therefore, we believe that the medicinal properties of *Cassia timoriensis* might have the potential to be developed as herbal products with antimicrobial properties in the future.

### 2.4. In Vitro Anti-Acetylcholinesterase Activity of Cassia timoriensis Flower Extracts 

Alzheimer’s disease (AD) is a progressive neurodegenerative disease indicated by low levels of acetylcholine (ACh) in the brain due to the activity of the acetylcholinesterase (AChE) enzyme. Lack of ACh in the brain has a great impact on short-term memory and learning. Preventing the enzyme from breaking down acetylcholine may ease some symptoms of AD [77]. The potential AChE inhibitory activity of *C. timoriensis* flowers was evaluated using Ellman’s method. Our results showed that at 200 µg/mL, all extracts except for the aqueous extract inhibited more than 90% AChE activity. Furthermore, the methanol and ethyl acetate extracts presented strong AChE activity inhibition with IC_50_ values of 6.40 ± 0.27 and 6.91 ± 0.38 µg/mL, respectively, followed by the hexane extract (IC_50_ 12.08 µg/mL) (Table 4). The potent inhibition of AChE activity by the ethyl acetate, methanol, and hexane extracts of *C. timoriensis* was in positive correlation to their high phenolic and flavonoid contents (Figure 1). Several mechanisms have been suggested for the anti-AChE activity of phenolic compounds, such as improving signal transmission in nerve synapses and increasing the concentration of ACh in synapses between cholinergic neurons [78,79,80]. 

*Cassia timoriensis* flowers have previously been shown to have anti-acetylcholinesterase activity [17]. However, variations in the inhibitory action of acetylcholinesterase within the same plant species have previously been observed [81,82]. These variations within the same plant species have been observed due to different phytoconstituents obtained from geographical regions but also according to seasons/periods of the year. The phytoconstituents of any plant part may vary both in quantity as well as quality depending on the soil, ground water level, stage of maturity of plant, and time of collection [81,82]. As a result, the IC_50_ value of the ethyl acetate fraction of *Cassia timoriensis* in this study (Table 4) is slightly different from that in the previously reported study [17]. 

In general, the *Cassia* genus is a promising source of anti-cholinesterase compounds. Few potential AChE inhibitors have been identified from *Cassia* species, such as anthraquinones (physcion, emodin, and alaternin), terpenoids (cassioates E and F), and 3-methoxyquercetin [17,83,84,85]. We postulate that the diverse secondary metabolites in the anti-AChE activity of *C. timoriensis* extracts, as seen in Table 1, may lead to the identification of potential compounds that inhibit AChE activity.

## 3. Materials and Methods

### 3.1. Materials (Chemicals)

Acetylcholinesterase (AChE) from *Electrophorus electricus* (electrical eels), type VI-S, 200–1000 unit/mg; substrate acetylthiocholine iodide (ATCI); sodium phosphate monobasic; and sodium phosphate dibasic were purchased from Sigma-Aldrich (St. Louis, MO, USA). The coloring agent 5,5-dithio-bis-[2-nitrobenzoic acid] (DTNB) and gallic acid were obtained from Acros (Geel, Belgium). Galantamine hydrobromide was obtained from Calbiochem (San Diego, CA, USA). Indomethacin, 2,2-diphenyl-1-picrylhydrazyl (DPPH), quercetin, zinc powder, and phosphate-buffered saline were also obtained from Sigma-Aldrich (St Louis, MO, USA). Wagner’s, Mayer’s, and Dragendorff’s reagents were obtained from R&M Chemicals (Essex, UK). Folin–Ciocalteu’s reagent and sodium hydroxide were obtained from R&M Chemicals (Essex, UK). Millon’s reagent for the detection of protein and sodium nitrite were obtained from Bendosen Laboratory Chemicals (Bendosen, Norway). Benedict’s solution for reducing sugar was obtained from PC laboratory reagents. Aluminum chloride was obtained from Quality reagent company (Auckland, New Zealand). Ferric chloride and sodium carbonate were obtained from Merck (Darmstadt, Germany). All solvents used were of analytical grade. 

### 3.2. Plant Collection and Identification

Fresh flowers were collected from the campus grounds of Universiti Sains Malaysia, Penang, in October 2019. The plant was identified and authenticated as *Cassia timoriensis* DC. by the Herbarium Deposition Department, Universiti Sains Malaysia. The voucher specimen (No. 11852) was deposited in the Herbarium of School of Biological Sciences, Universiti Sains Malaysia. The current taxonomy classification of this plant was referred to the Plant List website (www.theplantlist.com, accessed on 1 March 2020).

### 3.3. Plant Extraction and Fractionation

The flowers were dried in an oven at 40 °C for two days and then stored in an airtight container until further analysis. The dried flowers of *C. timoriensis* were ground into coarse particles and subjected to extraction using a simple maceration method. Briefly, the dried flowers of *C. timoriensis* (40 g) were extracted successively with continuous shaking for two days using 250 mL of different solvents with ascending polarities, namely *n*-hexane, ethyl acetate, methanol, and distilled water. The procedure was repeated three times to obtain the maximum yield of each fraction. All extracts were filtered and pooled accordingly and then evaporated under reduced pressure at 40 °C to yield solid residues of *n*-hexane extract (HE, 0.32 g), ethyl acetate extract (EE, 4.63 g), methanol extract (ME, 3.50 g), and aqueous extract (AE, 3.55 g). All extracts were kept in amber, airtight containers at 4 °C until further analysis.

### 3.4. Phytochemical Screening

Phytochemical screening of *C. timoriensis* extracts was performed to test for the presence or absence of bioactive constituents using standard protocols [18,19,20] to identify the secondary metabolites (flavonoids, alkaloids, saponins, tannins, and terpenoids) present in the HE, EE, ME, and AE (Table 5).

### 3.5. Antioxidant Capacity 

#### 3.5.1. Total Flavonoid Content (TFC)

The total flavonoid content of each extract was determined using the aluminum chloride colorimetric method with some adjustments [23]. Quercetin was used as a standard to construct the calibration curve. A series of dilutions (100, 200, 400, 600, and 1000 µg/mL) of each extract and standard were prepared using methanol as a solvent. An aliquot of 250 µL of each dilution was mixed with 1000 µL of distilled water, 75 µL of 5% sodium nitrite, and 75 µL of 10% aluminum chloride. After 5 min, 1 mL of 4% sodium hydroxide was added, and the volume was increased 2.5 mL using distilled water. After 15 min incubation at room temperature, the absorbance was measured at 415 nm using an Epoch Microplate Spectrophotometer (BioTek Instruments, Inc., Winooski, VT, USA). The assay was conducted in triplicate. The flavonoid content was estimated from the calibration curve, and the concentration of the flavonoids was quantified as mg quercetin equivalent (QE) per g dry extract weight.

#### 3.5.2. Total Phenolic Content (TPC)

The Folin–Ciocalteu method [86], with some modifications, was carried out to determine the total polyphenol content of the extracts. A series of dilutions (10, 20, 50, 100, 150, 200, 400, and 600 µg/mL) of the standard (gallic acid) were prepared to construct a calibration curve of gallic acid. The assay was performed by mixing 10 µL of each sample (1 mg/mL) with 50 µL of 10% Folin reagent followed by the addition of 60 µL of distilled water. A blank reagent was made with methanol. After 5 min incubation at room temperature, 80 μL of 7.5% sodium carbonate solution was added. Then, all samples were incubated in the dark for 30 min, and the absorbance was recorded at 765 nm using an Epoch Microplate Spectrophotometer (BioTek Instruments, Inc., Winooski, VT, USA). The assay was conducted in triplicate, and the TPC was quantified as mg gallic acid equivalent (GAE) per g of dry extract weight.

#### 3.5.3. Radical Scavenging Capacity

The antioxidant activity of *C. timoriensis* was measured using the DPPH method [24]. Briefly, a stock solution of each extract was prepared (1 mg/mL) using methanol. Then, a series of dilutions were prepared to obtain a solution at concentrations of 50, 25, 12.5, 6.25, and 3.125 µg/mL. A freshly prepared DPPH (2,2-diphenyl-1-picrylhydrazyl) solution was made by dissolving 4 mg of DPPH into 100 mL of methanol away from direct light. Then, using a 96-well plate, 150 µL of DPPH solution was mixed with 50 µL of different samples (50–3.125 µg/mL). Ascorbic acid solutions in methanol (50–1.562 µg/mL) were prepared and used as a positive control. After 30 min incubation, the absorbance was measured at 517 nm using a microplate reader (Epoch Microplate Spectrophotometer, BioTek Instruments, Inc., Winooski, VT, USA). A lower absorbance value indicates higher antioxidant activity of the sample. The % inhibition of the sample was calculated at a final concentration of 50 µg/mL. The results were expressed in IC_50_ values for samples that showed an inhibition percentage higher than 50%. The assay was conducted in triplicate for three consecutive days. 

The DPPH free radical scavenging ability at a concentration of 200 µg/mL was calculated using the following Equation (1):

(1) $$\%\;\mathrm{of}\;\mathrm{radical}\;\mathrm{scavenging}\;\mathrm{activity}=\frac{\mathrm{Abs}\;\mathrm{control}-\mathrm{Abs}\;\mathrm{sample}\;}{\mathrm{Abs}\;\mathrm{control}\;}\times 100$$

### 3.6. Anti-Inflammatory Activity 

Protein denaturation is considered a hallmark of inflammation. The anti-inflammatory potential of the plant extracts was evaluated using a heat-induced albumin denaturation assay [87,88]. The reaction mixture consisted of 1 mL of each plant extract at varying concentrations (100 and 200 µg/mL) or the reference compound, indomethacin (100 and 200 µg/mL), mixed with 200 µL of chicken egg albumin (fresh hen’s egg). The pH of the reaction mixture was calibrated to pH 6.4 using phosphate-buffered saline. The samples were incubated at 37 °C for 20 min, and then, the temperature was increased to 50 °C for 20 min. After incubation, the samples were immediately cooled on ice, and the turbidity was evaluated at 660 nm [89]. The assay was performed in triplicate. The percentage of inhibition of albumin denaturation was calculated using the following Equation (2):

(2) $$\%\;\mathrm{inhibition}\;=\frac{\left(\mathrm{Abs}\;\mathrm{control}-\mathrm{Abs}\;\mathrm{sample}\right)}{\mathrm{Abs}\;\mathrm{control}}\times 100$$

### 3.7. Inhibition of Acetylcholinesterase Activity

The in vitro potential of acetylcholinesterase inhibitory activity was performed spectrophotometrically using Ellman’s method [90,91]. The assay was conducted in a 96-well plate with a total assay mixture volume of 200 µL. Galantamine was used as the positive control. In a 96-well plate, an aliquot of 1 µL of extract (40 mg/mL DMSO) was mixed with 179 µL of 0.05 mM phosphate buffer, and 10 µL of 0.5 U/mL AChE (AChE from *Electrophorus electricus* (electrical eels), Type VI-S, 200–1000 unit/mg protein) was added to the designated wells. After 15 min incubation at 25 °C, 10 µL of equal amounts of 14 mM acetylthiocholine iodide (ATCI) substrate and 10 mM 5,5′-dithiobis-(2-nitrobenzoic acid) (DTNB) as a color indicator was added into each well and incubated at 25 °C for 30 mins to initiate an enzyme reaction. The absorption was measured at 415 nm using a Promega Glomax^®^ Multi Plus Reader (Promega, Sunnyvale, CA, USA). Each run was carried out in triplicate on three different days to determine the percentage of inhibition at 200 µg/mL. The % inhibition was determined using Equation (3):

(3) $$\%\;\mathrm{Inhibition}=\frac{\mathrm{Abs}\;\left(-\right)\mathrm{ve}\;\mathrm{control}-\mathrm{Abs}\;\mathrm{test}\;\mathrm{sample}}{\mathrm{Abs}\;\left(-\right)\mathrm{ve}\;\mathrm{control}}\times 100$$

Afterward, the IC_50_ value for each sample showing AChE inhibitory activity of 50% or more was determined.

### 3.8. Statistical Analysis

All measurements were performed in triplicate, and the results were expressed as mean ± SD. The experimental results were further analyzed using MS Excel and GraphPad Prism 8 statistical software (v. 8.0.2(263), San Diego, CA, USA).

## 4. Conclusions

In this study, the qualitative phytochemical analysis showed that all extracts of *C. timoriensis* flowers are rich in secondary metabolites, mainly comprising of flavonoids, tannins, coumarins, steroids, and terpenoids known to have a wide range of biological activities. In addition, the quantitative phytochemical analysis showed that ethyl acetate and methanol extracts possess the highest TPC (527.43 ± 5.83 and 321.746 ± 11.33 mg GAE/g DW, respectively) and TFC (851.83 ± 10.08 and 493.92 ± 9.27 mg QE/g DW, respectively). The ethyl acetate and methanol extracts of *C. timoriensis* exhibited great antioxidant (IC_50_ = 20.12 ± 0.12 and 34.48 ± 0.07 µg/mL, respectively), anti-inflammatory (92.50% ± 1.38 and 92.22% ± 1.09, respectively), and anti-cholinesterase (IC_50_ = 6.91 ± 0.38 and 6.40 ± 0.27 µg/mL, respectively) activities, probably due to their high phenolic and flavonoid contents. Given these data, more extensive research is needed to investigate the chemical constituents of ethyl acetate and methanol extracts of *C. timoriensis*, which may be responsible for the anti-Alzheimer effect.

## Figures and Tables

**Table 1:** Screening of phytochemical content of four different extracts of Cassia timoriensis flower.

No.	Class	Test	HE	EE	ME	AE
1	Alkaloids	Mayer’s test	-	-	-	-
		Wagner’s test	-	-	-	-
		Dragendorff’s test	-	-	-	-
2	Flavonoids	Alkaline reagent test	+	+	+	-
		Zn/HCL reduction test	+	+	+	-
3	Tannins	Ferric chloride test	+	+	+	+
4	Saponins	Frothing test	-	-	-	+
5	Cardiac glycosides	Keller–Killiani test	-	-	-	-
6	Anthraquinones glycoside	Borntrager’s test	-	+	-	-
7	Steroids	Liebermann–Burchard test	+	+	+	-
		Salkowski test	+	+	+	+
8	Terpenoids	Modified Salkowski test	+	-	+	-
9	Coumarins	-	+	+	+	-
10	Quinones	-	-	-	-	-
11	Proteins	Millon’s test	-	+	+	+
12	Carbohydrates	Benedict’s test (reducing sugar)	-	+	+	+

(+) indicates the presence of a compound class, (-) indicates the absence of a compound class. HE: hexane extract; EE: ethyl acetate extract; ME: methanol extract; AE: aqueous extract.

**Table 2:** Total phenolic content, total flavonoid content, and antioxidant activity of Cassia timoriensis flower extracts.

Sample	TPC mg GAE/g DW	TFC mg QE/g DW	Antioxidant Activity (DPPH Assay)	
			% Inhibition *	IC_50_ (µg/mL)
*n*-Hexane extract	136.36 ± 9.58	300.58 ± 10.78	45.18 ± 0.51	54.08 ± 0.78
Ethyl acetate extract	527.43 ± 5.83	851.83 ± 10.08	97.80 ± 0.29	20.12 ± 0.12
Methanol extract	321.75 ± 11.33	493.92 ± 9.27	71.74 ± 0.39	34.48 ± 0.07
Aqueous extract	31.05 ± 7.94	61.83 ± 9.10	12.18 ± 2.58	-
Ascorbic acid	-	-	98.73 ± 0.25	20.22 ± 0.03

Data are presented as mean ± SD, with *n* = 3; * % Inhibition was measured at a final concentration of 50 µg/mL. DPPH: 2,2-diphenyl-1-picrylhydrazyl; GAE: gallic acid equivalent; QE: quercetin equivalent; DW: dry weight.

**Table 3:** In vitro inhibition of protein denaturation by Cassia timoriensis flowers.

Sample	Concentration (µg/mL)	% Inhibition **
*n*-Hexane extract	100	43.13 ± 2.63
	200	85.25 ± 2.50
Ethyl acetate extract	100	92.38 ± 0.74
	200	92.50 ± 1.38
Methanol extract	100	89.45 ± 1.25
	200	92.22 ± 1.09
Aqueous extract	100	36.76 ± 1.50
	200	87.16 ± 2.02
Indomethacin	100	90.04 ± 0.87
	200	91.15 ± 0.32

** Data are presented as mean ± SD (*n* = 3).

**Table 4:** The activity of Cassia timoriensis against acetylcholinesterase enzymes.

Sample	% Inhibition *	IC_50_ (µg/mL)
Galantamine	98.64 ± 0.01	1.33 ± 0.03
Aqueous extract	38.32 ± 0.09	-
Methanol extract	96.55 ± 0.02	6.40 ± 0.27
Ethyl acetate extract	96.87 ± 0.05	6.91 ± 0.38
*n*-Hexane extract	92.35 ± 0.014	12.08 ± 0.95

Data are presented as mean ± SD (*n* = 3); * % Inhibition at 200 µg/mL.

**Table 5:** Qualitative phytochemical tests used for the screening of Cassia timoriensis extracts.

No.	Class	Test	Method	Positive Result	Ref.
1	Alkaloids	Mayer’s test	A few milligrams of each extract were dissolved individually in dilute HCL and filtered. Then, the filtrates were separately treated with Mayer’s, Wagner’s, and Dragendorff’s Reagents to test for the presence of alkaloids.	Turbidity or creamy precipitate	[19]
		Wagner’s test		Yellow–brown precipitate	[19]
		Dragendorff’s test		Turbidity or orange–red precipitate	[19]
2	Flavonoids	Alkaline test	About 2 mL of 20% NaOH solution was added to 1 mL of alcoholic solution of each plant extract individually.	Observation of intense yellow color	[19]
		Zn/HCl test	A pinch of zinc dust added to 2 mL of the alcoholic solution of sample. Then, a few drops of concentrated HCL were added slowly.	Observation of pink to red color	[20]
3	Tannins	Ferric chloride test	About 10 mg of the extracts was boiled in 10 mL of water in a test tube and then filtered. Then, a few drops of 1% ferric chloride wew added to the filtrate.	Hydrolysable tannins give bluish-black color, while condensed give brownish-green color	[19]
4	Saponin	Frothing test	A few milligrams of each extract were mixed separately with 5 mL of distilled water and mixed vigorously.	Persistent foam	[19]
5	Cardiac glycoside	Keller–Killiani test	About 3 mg of each extract was dissolved in 3 mL of concentrated acetic acid. Then, one drop of 5% FeCl_3_ solution was added, followed by few drops of concentrated sulphuric acid.	A reddish-brown ring forms at the interface	[18,19]
6	Anthraquinone glycoside	Borntrager’s test	A few milligrams of each extract were treated with dilute HCL and boiled for 5 min, cooled, and shaken with an equal volume of chloroform, benzene, or any other organic layer; then, the organic layer was separated and treated with ammonia.	Pink to red color in aqueous alkaline layer	[19]
7	Steroids	Salkowski’s test	A few milligrams of sample were treated with chloroform and filtered. The filtrates were then treated with a few drops of concentrated sulfuric acid.	Greenish-yellow color indicates the presence of steroids	[18,19]
		Liebermann–Burchard test	About 2 mg of each extract was dissolved in acetic anhydride, heated, and cooled before adding 1 mL of concentrated sulphuric acid along the test tube’s sides.	Green color indicates the presence of steroids nucleus	[18,19]
8	Triterpenoids	Modified Salkowoski’s test	About 1 mL of each of the four extracts was added to 1 mL of chloroform and filtered to clarify the solution, followed by dropwise addition of a few drops of concentrated sulphuric acid at the wall side of test tube.	Observation of reddish-brown color	
9	Coumarins	-	To 2 mL of each extract, a few drops of 10% alcoholic NaOH were added.	Observation of yellow color	[19]
10	Quinone	-	To 1 mL of each extract, a few drops of NaOH were added.	Observation of red or blue green color	[19]
11	Protein	Million’s test	A few drops of Million’s reagent were added to 2 mL of each sample and mixed.	Red color or precipitate indicated the presence of protein	[19]
12	Carbohydrate	Benedict’s test	A few drops of Benedict’s reagent were added to an aqueous solution of each plant extract and mixed.	Observation of orange–red color	[19]

## Data Availability

The data presented in this study are available in this article.

## Sample Availability

Samples of the compounds are not available from the authors.
